# Aberrant Crypt Foci: The Case for Inclusion as a Biomarker for Colon Cancer

**DOI:** 10.3390/cancers2031705

**Published:** 2010-09-16

**Authors:** Michael J. Wargovich, Vondina R. Brown, Jay Morris

**Affiliations:** Department of Cell and Molecular Pharmacology and Experimental Therapeutics, Hollings Cancer Center, Medical University of South Carolina, Charleston SC 29425 USA; E-Mails: browv@musc.edu (V.B.); morrisjl@musc.edu (J.M.)

**Keywords:** aberrant crypt foci, biomarker, colon cancer, chemoprevention

## Abstract

Aberrant crypt foci (ACF) are one of the earliest histopathological manifestations of colon cancer. In this review, we critically present the molecular, cellular, histopathological, and chemopreventive evidence that ACF are relevant biomarkers for colon cancer. The laboratory and clinical evidence are highly suggestive that ACF are in the pathway leading to colon cancer, but not all ACF will do so. The possible fate and outcome of ACF in the progression toward colon cancer may be dependent on a number of features that define their predictive power for the prevention or progression of cancer.

## 1. Historical Background: Discovery of Aberrant Crypt Foci

Colorectal cancer is still the second most common cause of cancer death in the United States [1]. The development of colorectal cancer depends on many lifestyle related factors in addition to genetic factors that influence the digestive tract [2]. Finding the point at which normal colonic epithelium becomes neoplastic, hyperplastic, dysplastic, or an early indicator of disease is of interest to many research groups. Evidence is mounting supporting the idea that aberrant crypt foci (ACF) are colon cancer precursors [3] whose size and numbers directly correlate with the risk of developing colon cancer. 

Aberrant crypt foci were first discovered by Bird in 1987 [4]. Treating mice with the carcinogen azoxymethane (AOM) induced the growth of colonic crypts that were larger, thicker and darker staining than normal crypts when visualized with methylene blue [4]. Additionally, the aberrant crypts seen in rodent models have distorted, slit-like luminal openings, and a noticeably thickened epithelia [5]. Aberrant crypt formation in rodent models is dose responsive to AOM. The size and number of crypts per focus as well as the size of the focus increases with increasing doses of carcinogen [6]. 

Aberrant crypts were first observed in the surrounding normal colonic mucosa of patients with colon cancer in 1991 [7]. The crypt clusters found in human mucosa appear raised from the normal mucosal surface of the colon [7]. Due to the rapid turnover of intestinal and colonic cells under normal conditions, it is expected that aberrant crypts would replicate at the same rate, if not faster than normal crypts. However, in humans there is conflicting evidence as to how much of an increase in replication exists, if any [8]. This inconsistency can be due to many factors, most notable difference between the methods of sampling and analysis in various studies determining colonic epithelial cell proliferation [9,10,11,12,13,14,15,16,17]. Aberrant crypt replication is essentially identical to that of normal crypts with the replication process starting at the bottom of the crypt pushing cells upward and outwards to form new colonic crypts in addition to replenishing the cells in the original crypt [18]. This is a budding and branching process, known as crypt fission, which forms larger sized foci over time [18,19]. This process does, however, occur at an increased rate in various disease states of the bowel [8,20].

Since ACF are considered putative precursors for cancer, interventions and therapeutics are targeted to alter this stage or earlier in the disease progress to either halt disease progression, reverse it, or prevent ACF formation. ACF formation accompanies changes in the morphology of colonic crypts in both benign diseases of the bowel and colon cancer. Since ACF formation is currently the earliest noted change visible with only a microscope, ACF can be used as a biomarker for disease states, including colon cancer.

## 2. ACF as a Relevant Biomarker for Colon Cancer: Histological Evidence

ACF were first reported in the colon epithelium of rodents treated with chemical carcinogens [5,21]. ACF are single to multiple crypt clusters of abnormally staining crypts after short-term staining with either methylene-blue or indigo-carmine solutions and fixation with either buffered formalin or alcohol-based fixatives [4,22]. Care is taken to fix rodent colon in a flat position, so that the entire topography of the organ is evident and to allow for observation of the characteristics features of ACF such as darker staining, increase size and slit like lumens [5,7,18,23]. ACF are readily visible usually with the aid of a dissection microscope at a magnification of at × 40.

There is a considerable wealth of literature describing the key histopathological signatures of ACF, and categorizing them in human has met with considerable controversy [23,24,25,26,27,28]. Microscopically, a distinction has been made between dysplastic ACF and non-dysplastic ACF (often including serrated hyperplastic ACF). Table 1 summarizes characteristics defining these three types of ACF. Studies in animal models have more often than not identified ACF that are more reflective of the dysplastic variety [29]. This may be the result of the employ of robust chemical carcinogens to induce ACF and colon cancer in rodents, or due to the use of transgenic animals expressing genes believed to be mutated in human colon cancer. 

**Table 1:** General and Histological Signatures of Rodent Aberrant Crypt Foci (ACF).

Characteristic		Type of ACF		
	Non-Dysplastic	Hyperplastic	Dysplastic	Reference
Darker staining	Yes	Yes	Darkest	[29,30]
Size	Increased	Increased	Increased	[29,31]
Topography	Raised	Raised	Raised	[31]
Diameter	Widest	Wide	Wide	[21,30,32]
Dilated lumen	Yes	Mixed	Thickened and closing	[30,31]
Pericryptal area	Serrated	Mixed	Non-serrated	[6,21,33]
Mucin status	Present	Mildly depleted	Depleted	[33,34,35,36,37]
Polarity	Ordered	Mixed	Lost	[29]
Nuclear morphometry	Round & non-stratified	Mixed	Oval & stratified	[21,29,30]
Proliferation pattern	Lower two-thirds of crypt	Progression to upper crypt	Full progression throughout crypt	[8,38]

**Figure 1:**
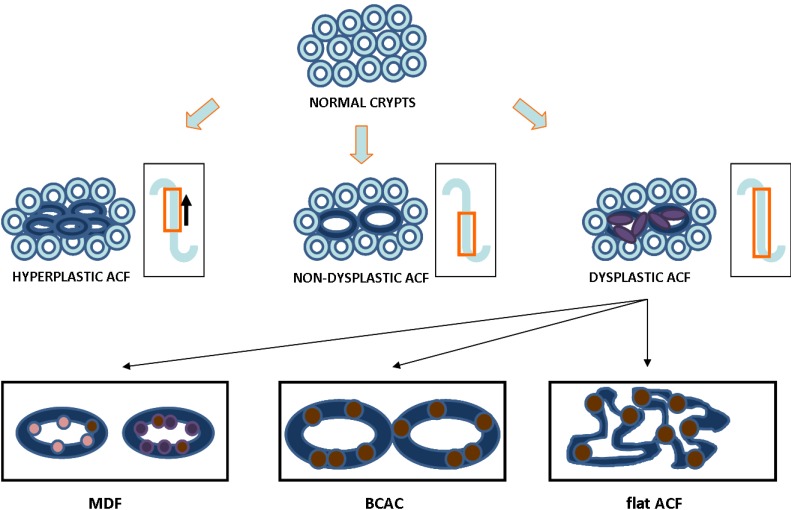
Three ACF types and possible pathways in colon carcinogenesis. The inset shows the speculated pattern of proliferation zone in the crypt of subtypes of ACF. Hypothetical scheme for the transition of crypts from normal to dysplastic ACF showing nuclear morphometry (dark blue is ACF; purple is stratified nucleus). Three types of dysplastic ACF reported in the literature: mucin depleted foci (MDF, goblet cells in pink are lost and replaced by stratified nuclei in purple; brown represent β-catenin which can accumulate in MDF), β-catenin accumulated crypts (BCAC, brown denoted nuclear β-catenin within the crypts) and flat-dysplastic ACF (flat ACF, brown is β-catenin within highly dysplastic and pleiomorphic crypts).

Published research has also defined ACF in other contexts: ACF that overexpress β catenin (beta catenin accumulated aberrant crypts, or BCAC) and mucin-depleted foci (MDF) [39,40]. Even flattened-dysplastic ACF have been described, and in most instances, these ACF also overexpress β catenin [37,41] (Figure 1). It is possible that other histopathological lesions could well associate with future risk factors for colon cancer. These include MDF and BCAC without the signature features of aberrant crypt foci. 

The published evidence is consistent with the hypothesis that dysplastic ACF are precursors for colon cancer, at least based on the histopathology. In presenting the case for inclusion as a biomarker for colon cancer, some ACF evidence features common with adenomas: they are depleted in mucin, have a monoclonal origin, they demonstrate superficial to deep dysplasia; they are hyperproliferative, and evidence nuclear staining for β catenin [21,25,40,42,43,44]. Arguably, this case can best be made for those advanced ACF seen in carcinogen-based or transgenic animal models for colon cancer. The available evidence in humans, as we will see, is more controversial.

## 3. ACF as a Relevant Biomarker for Colon Cancer: Molecular and Cellular Evidence

ACFs can be used as an identifiable, early marker for onset of colon carcinogenesis [29]. With the advent of new molecular biology assays, further characterization of ACF genetic profiles to assess their risks of progression to cancers are possible [44]. These categories consist of epigenetic silencing, genetic mutation, microsatellite instability and loss of heterozygosity (Table 2) [45,46,47,48,49,50,51,52,53,54,55,56,57,58,59,60,61,62,63,64,65,66,67]. Epigenetic silencing of genes involved in cancers has been an area of intense research over the last decade [63]. These alterations are prevalent in ACFs with a tendency to occur in the beginning and early stages of formation [64]. Unlike epigenetic silencing, genetic mutations involve changes in the coding regions of a gene. The formation of ACFs is a multistep process and there are several notable mutations that occur at varying times within the crypt development (Table 2). Assessment of the mutational status of these genes within ACFs can help define the carcinogenic potential of crypts, although some characteristics are better suited to be studied at later stages of polyp development [22]. One report showed [10] an occurrence of mutant p53 in 42% of ACF but more recent studies have shown that p53 loss does not highly occur in ACF but rather more frequently in later stages of polyp development [22]. A further aspect to characterize ACF is the presence of microsatellite instability (MSI) [45,59,68]. MSI occurs during the early stage of ACF with a high degree of frequency [45,59,68]. Although MSI is a factor in Hereditary Nonpolyposis Colorectal Cancer (HNPCC) [59], and less so in sporadic colorectal carcinogenesis, it still may represent a diagnostic target in ACF formation. Loss of heterozygosity (LOH) has commonly been affiliated with the later stages of carcinogenesis and not observed as frequently with ACF formation, but recent work has shown that LOH in certain areas could occur possibly before APC mutations [62]. Although we have listed a few factors here, there are numerous other genetic components of ACFs. Together with the histopathology analysis, the molecular signatures strengthen the evidence that ACF maybe useful as a colon cancer biomarker. These characteristics may define future research to determine the exact role of ACFs in the pathway of carcinogenesis. 

**Table 2:** Molecular characteristics of ACFs.

Molecular Category	Genetic Components^a^	Approximate occurrence in ACF formation^b^	Likelihood of progression to adenocarcinoma	Reference
Epigenetic Silencing	*MINT31*	34%	Majority	[49]
	*SFRP1*	93%	Possible	[65]
	*SFRP2*	87%	Possible	[65]
Genetic Mutation	*APC*	Less than 10%	Majority	[69]
	*β-catenin*	0	Possible	[69]
	*K-ras*	40%	Majority	[46]
	*p53*	Less than 10%	Possible	[46]
Microsatellite Instability^c^	*hMLH 1*	Less than 10%	Probable	[59]
			

a. Selected genetic targets; b. Represent analysis of ACF from human tissue; c. Patients were screen for HNPCC cancerr type [59].

## 4. ACF as a Relevant Biomarker for Colon Cancer: Chemoprevention Studies

Over 300 agents have been tested in rodents for colon cancer chemoprevention efficacy [30,70,71]. However, the choice of animal species, method of induction of colon neoplasia, and timeframe of exposure to putative chemoprotectants has varied considerably. The first question is to decide whether to use mice or rats. The rat has obvious advantages in terms of its size and ease of manipulation of its colon. The mouse has the advantage of using genetic knock-outs and expression of transgenes of mechanistic interest. Most agents that have been screened in animal models for chemopreventive efficacy have also been tested in carcinogen-induced systems, which are foreshortened variations of full-term colon carcinogenesis protocols. While the relevancy of using a carcinogen-based system is still debatable, researchers have relied on the histopathological similarity of either the dimethylhydazine or azoxymethane methods of inducing ACF [25,29,72]. Yet, researchers have employed an array of different carcinogens, including MNU (*N*-methyl-*N*-nitrosourea), IQ (2-amino-3-methylimidazo[4,5-f] 95quinoline), PHiP (2-amino-1-methyl-6-phenylimidazo [73]spyridine), and others [73,74,75]. Each has its own merits and liabilities. After deciding which animal to use, the inducing agent and its dose, the next step is to determine the time course in which the test agent will be delivered. The most common protocol is to test the agent during the exposure to the carcinogen (approximately a five week experiment), or after carcinogen dosing is completed (approximately an eight week experiment). To investigate the efficacy of test agents on ACF with higher crypt multiplicities and varying morphologies, the duration may need to be extended to as much as 24 weeks [29]. A database maintained (http://corpet.net/min) by Dr. Denis Corpet (INRA, France) lists the results on nearly all experiments in rodent ACF assays that have been published and is updated frequently [76]. An inspection of the database leads us to propose a standard protocol for rodent chemoprevention efficacy assays using ACF as the endpoint. The number of animals per group is determined by power calculations following individual laboratory experience, but often 10 mice per group are sufficient. The INRA database lists agents that have been tested in the rodent ACF assays sorted by a number of parameters, with potency being the most informative and of these, the category of phytochemicals evidences many of the more potent agents. From these data and the experience of our laboratory, we propose recommendations for standardized protocols for chemopreventive efficacy testing in rodents. These are shown in Table 3.

Overall, the published evidence is consistent with the idea that agents that prevent ACF will, with high concordance, also predict prevention of cancer in full length carcinogenesis models.

**Table 3:** Recommended Standardized Protocols for Chemoprevention Efficacy Testing in Colorectal Cancer Animal Models.

Rodent & Species	Method of Induction	Dose^a^	Route	Timeframe of Exposure of Test Agent^b^
Mouse: CF-1	Azoxymethane	2 × 10	i.p.	Initiation: 1 or 2 weeks before first AOM dose and ending after the second AOM dose
				Post-Initiation: 8 weeks after final AOM dose
Rat: F344	Azoxymethane	2 × 15	i.p.	Initiation: 4 weeks after final AOM dose
				Post-Initiation: 8 weeks after final AOM dose

a. Frequency of weekly injections and dose of AOM in mg/kg, *i.e.*, two separate injections a week at 10 mg/kg or 15 mg/kg; b. The agent is usually administered in a semi-synthetic diet such as AIN76A or AIN93G.

## 5. ACF as a Relevant Biomarker for Colon Cancer: Clinical Evidence

Molecular tools now can more accurately predict ACF progression animal models; however, the similar characterization of ACF in humans remains ambiguous. The problem arises due to high amount of variability in methods and criteria when endoscopically assessing tissues for ACF prevalence [24,25,77]. High-Magnification-Chromoscopic Colonoscopy (HMCC) allows for better visual scanning of ACF with little to no discomfort to the patients [78]. This increased resolution increases diagnostic efficiency of identifying ACFs and determining morphology [77]. Although technology is improving the ability to detect and classify ACF, there is still controversy surrounding ACFs as a putative marker for CRC [27,28]. Patient access to these research-driven endoscopes and increased time for identification and classification are just some of the difficulties in a clinical ACF study [27]. Sampling of ACF from the human colon is still problematic.

Despite the time and visual obstacles, ACF have been utilized as markers of CRC in numerous studies. These ranged from clinical trials testing experimental therapeutics in HNPCC patients to documentation of ACF in patients with sporadic colon carcinomas [49,59,78,79]. The ever changing environment and constant turnover of cells within the intestinal lumen makes tracking ACF development and tissue progression difficult, and serial studies are not feasible. Furthermore, not all ACF develop into adenomas and within some individuals molecular characteristics of ACF vary from a high correlative pattern of possible progression to cancer to characteristics more reminiscent of healthy, and normal mucosal tissue [27,45,78]. As shown in Table 2, assessing the status of key genetic factors of ACF, following identification by visual means, improves the likelihood of finding problematic ACF. This combinatorial evaluation shows that key molecular factors of non-dysplastic ACF are less distorted compared to dysplastic ACF [24,78,79,80]. For example immunohistochemistry can assess differences in Ki-67 amounts between non-dysplastic and hyperplastic ACF [79]. Alterations of these molecular factors have been shown to be prominent characteristics of colon carcinomas inferring that dysplastic ACFs may indeed have a higher correlation of progression to carcinomas. The use of higher powered microscopic identification and the prevalence of uncomplicated, quick dissection and molecular diagnosis of tissues will alleviate some limitations in the use of ACF as biomarkers. 

In conclusion the use of ACF as a bona fide biomarker in human chemoprevention trials remains circumstantial [78,79]. However, a subset of ACF grossly presenting with dysplasia, further characterized by alteration in genetic pathways controlling cell proliferation, are potentially useful as markers to assess high risk individuals, future risk in asymptomatic individuals, and present targets for chemotherapeutic and chemopreventive compounds. 

## 6. Conclusions

The summary of the experimental evidence is very supportive of the hypothesis that ACF are a relevant biomarker for colon cancer. Our conclusions are based on the evidence that ACF demonstrate histological similarities to colon adenomas and adenocarcinomas, and share key molecular defects with at least some colon tumors. The evidence is more circumstantial for ACF in humans, as our ability to track their growth and change through the development of cancer is very limited. With the development of cancer stem cell discoveries, we may soon be able to recapitulate the development of ACF from single stem cells and watch their progression to overt tumors.
